# Ras Association Domain Family 1A Gene Promoter Methylation as a Biomarker for Chronic Viral Hepatitis C-related Hepatocellular Carcinoma

**DOI:** 10.7759/cureus.45687

**Published:** 2023-09-21

**Authors:** Abla A Abou Zeid, Eman T El-Sayed, Jylan K Ahdy, Marwa R Tawfik

**Affiliations:** 1 Clinical and Chemical Pathology, Alexandria Faculty of Medicine, Alexandria University, Alexandria, EGY; 2 Clinical and Chemical Pathology, Al Ramel Pediatric Hospital, Ministry of Health, Alexandria, EGY; 3 Hepatobiliary Unit, Internal Medicine Department, Alexandria Faculty of Medicine, Alexandria University, Alexandria, EGY

**Keywords:** methylation-specific pcr, rassf1a gene, chronic viral hepatitis c, hepatocellular carcinoma, serum biomarker

## Abstract

Background

One of the most prevalent aberrant epigenetic modifications found in hepatocellular carcinoma (HCC) is abnormal DNA methylation. Our study aimed to evaluate serum Ras association domain family 1A (RASSF1A) gene promoter methylation in patients with chronic viral hepatitis C (HCV)-associated liver cirrhosis with and without HCC as a potential new marker for the early detection of HCC.

Methodology

The 60 participants who participated in the trial were divided into the following three groups: 20 patients with newly diagnosed primary HCC on top of HCV-related liver cirrhosis, 20 patients with HCV-related liver cirrhosis, and 20 age- and sex-matched healthy individuals as a control group. All participants underwent methylation-specific polymerase chain reaction testing to detect the blood level of the RASSF1A gene’s methylated promoter.

Results

Methylated RASSF1A was found in 30% of patients with liver cirrhosis caused by HCV and in 65% of patients with HCC, but not in any of the controls. It was discovered that the serum methylation RASSF1A had an accuracy of 82.50% and an area under the curve (AUC) of 0.825 for separating HCC patients from healthy controls. With an AUC of 0.675 and an accuracy of 67.50%, it was able to differentiate patients with HCC from those with HCV-related liver cirrhosis. Additionally, there was no statistically significant association between RASSF1A methylation status and HCC mass size (p = 0.449).

Conclusions

Serum RASSF1A promoter methylation status detection could be useful for detecting HCC early, especially in high-risk individuals such as those with HCV.

## Introduction

Liver cancer is among the leading causes of cancer deaths globally. The most common causes of liver cancer include hepatitis B virus (HBV) and hepatitis C virus (HCV) infection and alcohol use. Cases of incident liver cancer increased by 75% between 1990 and 2015. The most common type of primary liver cancer globally is hepatocellular carcinoma (HCC) [1].

Important geographical differences in HCC incidence have been noted globally which may be attributed to differences in exposure to different risk factors, especially viral hepatitis [1]. The incidence in Egypt has increased during the last decade dramatically. This may be because Egypt is estimated to have the highest prevalence of HCV in the world [2,3]. The American Association for the Study of Liver Disease and the European Association for the Study of the Liver recommended regular follow-ups for the high-risk group by abdominal ultrasound every six months as a screening program for HCC in a trial for the early detection of the tumor mass. Diagnosis of HCC depends mainly on triphasic computerized tomography (CT) of the abdomen. Serum alpha-fetoprotein (AFP) assessment as a tumor marker is no longer recommended as a screening tool or diagnostic method for HCC [4,5]. About one-third of patients with HCC who have a small hepatic mass usually present with normal serum AFP levels. Serum AFP elevation is not specific to HCC only as its elevation is seen in several non-hepatic cancers. However, despite the great advances in the diagnostic modalities of HCC, the five-year survival of patients remains very low. Many recent studies now are concerned with the development of a new non-invasive marker that aids in the early diagnosis of HCC. Recent studies have reported that the molecular pathogenesis of HCC involves both aberrant genetics and/or epigenetic alterations. Among the most common aberrant epigenetic alterations studied in HCC is aberrant DNA methylation [6,7].

Aberrant DNA methylation is associated with several disease conditions including cancer. The detection of methylation changes offers a great opportunity for the early detection and diagnosis of cancer and may also allow the individualization of therapies. Determining a methylation signature may also predict the time to relapse and/or overall survival, which will have a great impact on individualized care regimens. The methylation status of many genes has been investigated in several cancer types [8,9].

The Ras association domain family 1A (RASSF1A) gene belongs to the Ras association gene family, which is located in the 3p21.3 locus which encodes regulatory proteins with tumor-suppressor functions. Cell cycle regulation, cellular adhesion, microtubule stabilization, cell motility, and apoptosis are all crucially influenced by RASSF1A, which is connected to the Ras signaling system. It is investigated in several cancer types, and RASSF1A promoter hypermethylation is observed in multiple human malignancies such as lung cancer (22.6%), pancreatic cancer (34.0%), gastric cancer (28.6%), breast cancer (28.2%), and colon cancer (28.3%) [9].

Some studies have proposed that methylation of the RASSF1A promoter may have an important role in the process of hepatocarcinogenesis in cirrhotic patients. These data may pave the way for the detection of RASSF1A promoter methylation status as a new marker for the early detection of HCC [10]. This study aimed to detect serum RASSF1A promoter methylation in patients with HCV-associated liver cirrhosis with and without HCC to evaluate its potential use as a marker for the detection of HCC in patients with chronic hepatitis C infection.

## Materials and methods

Study design and sampling

This study was conducted among 60 individuals. It was approved by the Institutional Review Board of the Alexandria Faculty of Medicine (reference number: 14-467) and adhered to the principles of the Helsinki Declaration. Each participant in the study provided informed consent. The patients were recruited from the inpatient ward and outpatient clinic of the Hepatobiliary Unit, Department of Internal Medicine at Alexandria University Hospital, Egypt. The control group participants were recruited from the minor surgery clinic at the same hospital. The participants were divided into the following three groups: group I: 20 patients with newly diagnosed primary HCC on top of HCV-related liver cirrhosis; group II: 20 patients with HCV-related liver cirrhosis; and group III: 20 healthy age- and sex-matched individuals as a control group. The exclusion criteria were previous anti-viral or systemic anti-cancer therapy, concomitant HBV infection, schistosomiasis, other malignancies, alcohol consumption, and obesity. All participants were randomly selected and recruited to each group according to the eligibility criteria by a simple random sampling method.

Data collection

All HCV-related cirrhotic patients, patients with HCC, and the control group were subjected to the following: full history including age and sex, history of alcohol consumption and smoking, family history of HCC (or other malignancies), and history of any other associated medical conditions such as non-alcoholic fatty liver disease or schistosomiasis. In addition, clinical presentation at the time of diagnosis included non-specific symptoms, symptoms of cirrhosis, or symptoms of distant metastasis. A thorough physical examination with stress on manifestations of advanced liver disease and HCC staging was done according to modified Barcelona Clinic Liver Cancer (BCLC), including tumor features, liver functions (according to modified Child-Pugh score), and patients’ performance status [11].

Laboratory Investigations

Complete blood count (CBC), liver test profile, and prothrombin time (PT); viral serological markers that involved HCV antibodies (HCV-Abs); HCV real-time polymerase chain reaction (PCR); hepatitis B surface antigen (HBsAg); and total hepatitis B core antibody (HBc-Ab total) were recorded. Additionally, testing for kidney functions and autoimmune markers and tumor markers, such as serum AFP and prostate-specific antigen, were performed as a part of the patients’ comprehensive assessment at the time of diagnosis. Molecular analysis with the detection of methylated RASSF1A in serum samples was performed using methylation-specific PCR (MSP) [12-14].

Radiological Investigations

Abdominal and pelvic ultrasonography were performed for the assessment of the liver (radiological diagnosis of liver cirrhosis), spleen, portal circulation, and ascites. Multiphasic contrast-enhanced CT and/or dynamic contrast-enhanced magnetic resonance imaging (MRI) were used to diagnose HCC and evaluate its imaging characteristics, including size, number of lesions, vascular involvement, and thrombosis.

Sample collection

In this study, 10 mL of whole blood was collected into five vacutainer tubes. First, 2 mL was collected in a vacutainer tube containing dipotassium ethylenediaminetetraacetic acid (K2EDTA) for CBC. Second, 2 mL was collected in a vacutainer tube containing a clot activator. Blood was left to clot at room temperature for 15 minutes, followed by centrifugation at 4,000 rpm for 10 minutes to separate serum. Then, 300 µL of the serum was collected in sterile Eppendorf and stored at -20°C for determination of AFP. The rest of the serum was used for the analysis of liver and kidney function tests and viral markers (HCV-Abs, HBsAg, total HBc-Abs). Third, 2 mL was collected in a vacutainer tube containing sodium citrate for PT. Fourth, 2 mL was collected in a vacutainer tube containing a clot activator. Blood was left to clot at room temperature for 15 minutes, followed by centrifugation at 4,000 rpm for 10 minutes to spin down blood cells. The supernatant was transferred to a microcentrifuge tube and centrifuged at 14,000 rpm for 10 minutes to ensure the complete removal of a cellular component. Serum aliquots were immediately frozen at -80°C to determine the methylation status of RASSF1A using MSP. Fifth, 2 mL was collected in a vacutainer tube containing K2EDTA (to separate plasma) for the detection of HCV by real-time PCR.

DNA Extraction

DNA was isolated from patients’ sera, using the QIAamp DNA Kits (Qiagen, Germany) following the manufacturer’s instructions.

Bisulfite Conversion

Bisulfite conversion was done using the EZ DNA Methylation-Gold kit (Catalog No D 5006) (Zymo Research, Irvine, CA, USA) according to the manufacturer’s instructions. Based on the principle that bisulfite treatment of DNA would convert unmethylated cytosine to uracil, methylated cytosine residues would remain unchanged. Thus, after bisulfite conversion, methylated and unmethylated DNA sequences would be easily distinguished by MSP.

Methylation-Specific PCR

The purified DNA was processed using an EpiTect MSP kit (Qiagen, Germany) following the manufacturer’s instructions. PCR was performed according to instructions. PCR conditions were as follows: thermal cycler Biometra (Biometra, Germany) was programmed according to the manufacturer’s instructions: The initial inactivation step was one cycle of 10 minutes at 95°C. The second step was 40 cycles at 94°C for 15 seconds, followed by at 55°C for 30 seconds, at 72°C for 30 seconds, and a final extension at 72°C for 10 minutes. After amplification, PCR products were evaluated by a 2% agarose gel electrophoresis, stained with ethidium bromide, and visualized under ultraviolet illumination. The interpretation of the results was easy according to the position of the lane where the band of expected molecular weight (169 bp) appeared. If it appeared in the methylated lane, then the sample had methylated RASSF1A alleles, but when it appeared in the non-methylated lane, the RASSF1A alleles were unmethylated.

Statistical analysis

Data were fed to the computer and analyzed using SPSS software package version 20.0 (IBM Corp., Armonk, NY, USA) [15,16]. Qualitative data were described using numbers and percentages. Quantitative data were described using range (minimum and maximum), mean, standard deviation, and median. The chi-square test was used to compare differences in categorical variables between several groups. Monte Carlo correction was used to compensate for chi-square when more than 20% of the cells had anticipated counts lower than 5.

The Kolmogorov-Smirnov test, Shapiro-Wilk test, and D'Agstino test, as well as the Histogram and QQ plot, were used to assess the normality of the distributions of quantitative variables.

Parametric tests were used if they showed a normal data distribution. Non-parametric tests were employed if the data were not normally distributed. For normally distributed data, a comparison between two groups was done using an independent t-test while more than two groups were analyzed using an F-test (analysis of variance test) and a post-hoc test (least significant difference) for abnormally distributed data. Comparison between different groups was done using the Kruskal-Wallis test and pairwise comparison was assessed using the Mann-Whitney test after p-value adjustment. The significance of the obtained results was judged at the 5% level. Receiver operating characteristic (ROC) curve analysis was performed to detect the diagnostic performance of different indices of HCC. The area under the curve (AUC), sensitivity, specificity, positive predictive value (PPV), and negative predictive value (NPV) were used to evaluate each index. For significant results, an AUC of 0.90-1 was deemed excellent, 0.80-0.90 good, 0.70-0.80 fair, 0.60-0.70 poor, and 0.50-0.60 fail.

Sample size calculation

A minimum required sample size of 20 HCC patients, 20 cirrhotic patients, and 20 healthy control patients achieved 80% power to detect a difference in the proportion of serum RASSF1A methylation. A similar study reported serum RASSF1A methylation occurred in 64.2% of patients with HCC compared to 17.5% among patients with liver cirrhosis and 0% in the control group [17]. The sample size was calculated based on an area under the curve (AUC) for the combination of serum RASSF1A methylation and AFP level (≥20 ng/L) of 0.876 to differentiate between HCC and liver cirrhosis. The sample size was calculated using R software pwr and pROC packages (R Foundation for Statistical Computing, Vienna, Austria) [18].

## Results

Demographic characteristics of the studied groups

The group of patients with HCC consisted of 18 (90.0%) men, while the group of cirrhotic patients consisted of 15 (75.0%) men, and the control group included 13 (65.0%) men. There was no statistically significant difference between the three groups regarding sex distribution (p = 0.209). No statistically significant difference was detected in mean age between the three groups (p = 0.223) Table 1.

**Table 1:** Comparison between the studied groups according to demographic data and routine laboratory investigations. *: Statistically significant atp ≤ 0.05. KWχ^2^: Kruskal-Wallis test. Significance between groups was assessed using the Mann-Whitney test. χ^2^: Chi-square test; MC: Monte Carlo test. F: F-test (one-way analysis of variance). Significance between groups was assessed using the post-hoc test (least significant difference). p1: P-value for comparing the HCC group and the cirrhotic group. p2: P-value for comparing the HCC group and the control group. p3: P-value for comparing the cirrhotic group and the control group. HCC: hepatocellular carcinoma; ALT: alanine aminotransferase; AST: aspartate aminotransferase; PT: prothrombin time; INR: international normalized ratio

	HCC (n = 20)	Cirrhotic (n = 0)	Control (n = 20)	Statistical significance	
Sex	No (%)			Test of significance	P
Male	18 (90)	15 (75)	13 (65)	χ^2^ = 3.534	^MC^p = 0.209
Female	2 (10)	5 (25)	7 (35)		
Age (years), mean ± SD	59.65 ± 5.96	55.65 ± 9.96	55.60 ± 8.69	F = 1.542	P = 0.223
Liver functions tests: median (minimum–maximum)					
Serum ALT (U/L)	51.0 (16.0–186.0)	40.5 (12.0–96.0)	26.0 (14.0–39.0)	KWχ^2^ = 15.787	P < 0.001^*^
	Significance between groups p1 = 0.107, p2 = 0.066, p3 < 0.001^*^				
Serum AST (U/L)	68.0 (7.0–177.0)	51.50 (13.0–2.0)	28.0 (14.0–35.0)	KWχ^2^ = 11.629	P = 0.003*
	Significance between groups p1 = 0.310, p2 = 0.039*, p3 = 0.001*				
Serum albumin (g/dL)	2.70 ± 0.75	2.95 ± 0.53	4.65 ± 0.53	F 59.820	P < 0.001*
	Significance between groups p1 = 0.202, p2 < 0.001*, p3 < 0.001*				
Serum total bilirubin (mg/dL)	1.30 (0.70–4.80)	1.10 (0.50–3.80)	0.80(0.40–1.10)	KWχ^2^ = 13.159	P = 0.001*
	Significance between groups p1 = 0.957, p2 = 0.001*, p3 = 0.004*				
Complete blood count					
Hemoglobin (g/dL), mean ± SD	10.56 ± 1.77	10.82 ± 2.19	12.27 ± 0.93	F 5.775	P = 0.005^*^
	Significance between groups p1 = 0.639, p2 = 0.003*, p3 = 0.010*				
Platelets (×10^3^/μL), mean ± SD	110.0 ± 39.14	119.80±56.33	297.75±67.87	F 71.966	P < 0.001*
	Significance between groups p1 = 0.580, p2 < 0.001*, p3 < 0.001*				
Coagulation profile: Mean ± SD					
PT (second)	15.44 ± 2.86	14.95 ± 1.39	11.30 ± 0.5	F 30.362^*^	P < 0.001*
	Significance between groups p1 = 0.405, p2 <0.001*, p3 <0.001*				
INR	1.52 ± 0.33	1.42 ± 0.19	1.0 ± 0.2	F 24.366*	P < 0.001*
	Significance between groups p1 = 0.932, p2 < 0.001*, p3 < 0.001*				

Distribution of the study sample according to the physical examination in patients with HCC and patients with cirrhosis

In the group of patients with HCC, nine (45.0%) were asymptomatic. Non-specific vague symptoms (abdominal pain, weight loss, easy fatigue, anorexia) were present in eight (40.0%) patients, while symptoms of cirrhosis (ascites, jaundice, hematemesis, palmar erythema, and spider angioma, gynecomastia, periumbilical collateral veins) were present in 11 (55.0%) patients. Extrahepatic metastases, paraneoplastic syndrome, or spontaneous rupture of HCC mass were absent. In the group of patients with cirrhosis, six (30.0%) were asymptomatic.

Radiological investigations

Triphasic CT abdomen revealed that 11 (55.0%) patients with HCC had a single hepatic mass and nine (45.0%) patients had multiple hepatic masses. The size of the hepatic mass ranged from 0.5 cm to 4.5 cm.

Clinical staging of patients with HCC was categorized as follows using the modified BCLC classification: four (20.0%) patients were in the very early stage (stage 0), seven (35.0%) were in the early stage (stage A), five (25.0%) were in the intermediate stage (stage B), two (10.0%) were in the advanced stage (stage C), and two (10.0%) were in the terminal stage (stage D).

Laboratory investigations

Median serum alanine transaminase, serum aspartate transaminase, serum total bilirubin, and coagulation profile (PT and international normalized ratio) were significantly higher among HCC and cirrhotic patients than control patients (p < 0.05). Serum albumin and CBC profile (hemoglobin, white blood cells, and platelets) were significantly lower among HCC and cirrhotic patients than among control patients (p < 0.05) (Table 1).

Viral and serum tumor markers

The mean viral load in patients with HCC was 2.26 × 10^6^ ± 0.60 IU/mL, while in cirrhotic patients, it was 2.43 × 10^4^ ± 0.62 IU/mL. A statistically significant difference was detected between the two groups regarding HCV viral load (p < 0.001).

Alpha-fetoprotein

**Figure 1:**
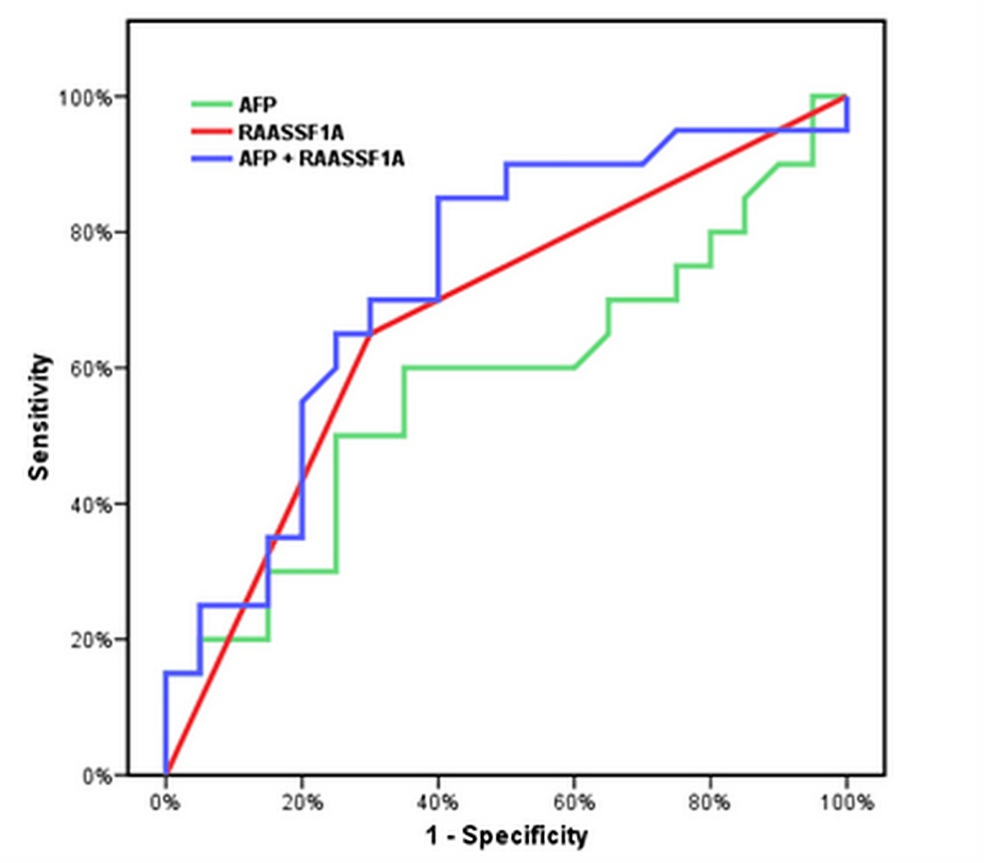
ROC curve for the combination of AFP and methylated RAASSF1A for diagnosis. AFP: 50% SN, 70% SP, 62.5% PPV, 58.33% NPV, AUC 0.573, and accuracy 60%. RASSF1A: 65% SN, 70% SP, 68.42 PPV, 66.67 NPP, AUC 0.675, and accuracy 67.5%. AFP + RASSF1A: 65.5% SN, 70% SP, 68.42 PPV, 66.67 NPP, AUC 0.685, and accuracy 67.5%. ROC: receiver operating characteristic; RAASSF1A: Ras association domain family 1A; HCC: hepatocellular carcinoma; AFP: alpha-fetoprotein; SN: sensitivity; SP: specificity; PPV: positive predictive value; NPV: negative predictive value; AUC: area under the curve

**Figure 2:**
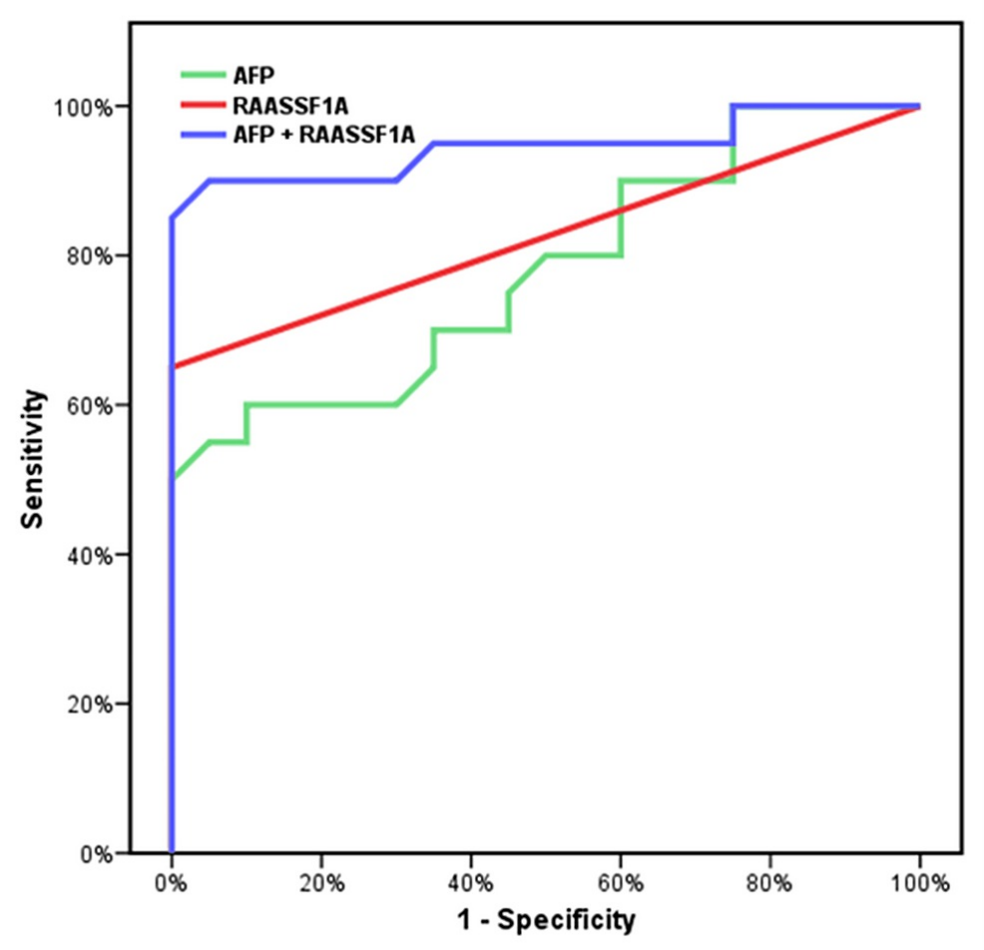
Diagnostic performance of the combination of AFP and methylated RAASSF1A AFP to detect HCC patients in the control group. AFP: 60% SN, 70% SP, 66.67 PPV, 63.64 NPP, AUC 0.779, and accuracy 65%. RASSF1A: 65.5% SN, 100% SP, 100 PPV, 74.07 NPP, AUC 0.825, and accuracy 82.5%. AFP + RASSF1A: 85% SN, 100% SP, 100% PPV, 86.96 NPP, AUC 0.945, and accuracy 92.5%. RAASSF1A: Ras association domain family 1A; HCC: hepatocellular carcinoma; AFP: alpha-fetoprotein; SN: sensitivity; SP: specificity; PPV: positive predictive value; NPV: negative predictive value; AUC: area under the curve

In the group of patients with HCC, AFP values varied from 59.0 to 1,210.0 ng/mL, with a median value of 446.0 ng/mL. On the other hand, it ranged from 6.50 to 45.0 ng/mL with a median value of 20.0 ng/mL in the cirrhotic group. AFP levels in the control group varied from 5.0 to 10.0 ng/mL, with a median value of 8.0 ng/mL. Both cirrhotic patients and healthy individuals showed a statistically significant difference (p3 = 0.001), as did the group of HCC patients and the control group (p2 = 0.001). Additionally, a statistically significant difference between the HCC patient group and the cirrhotic patient group was found (p10 = 0.001). ROC curve analysis was performed to evaluate the diagnostic performance of serum AFP in detecting HCC in cirrhotic patients. At a cut-off value of 17 ng/mL, AFP had a sensitivity of 50.0%, specificity of 70.0%, PPV of 62.5%, NPV of 58.33%, and accuracy of 60.0%. The AUC was 0.573 (Figure 1). Another ROC curve analysis was done to evaluate its diagnostic performance in the detection of HCC in the control group. At a cut-off value of 6 ng/mL, AFP sensitivity was 60.0%, specificity was 70.0%, PPV was 66.67%, NPV was 63.64%, and accuracy was 65.0%. The AUC was 0.779 (Figure 2).

Detection of the methylation status of RASSF1A in serum samples of patients

**Figure 3:**
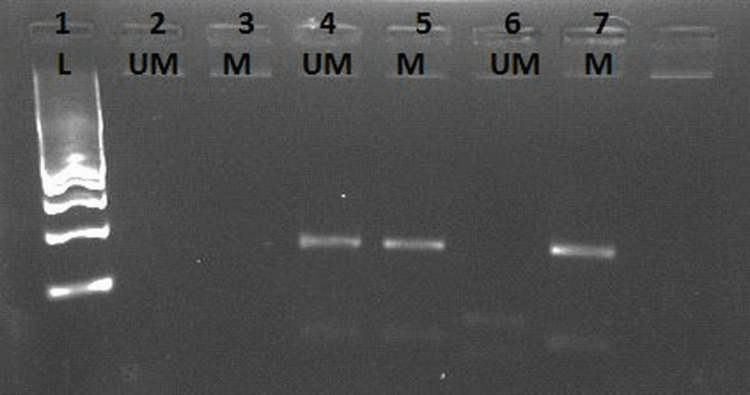
Analysis of the methylation status of RASSF1A in the serum of patients with HCC. Lane 1: Molecular weight marker (100 bp ladder). Lane 2: Unmethylated no template control (NTC). Lane 3: Methylated NTC. Lanes 4 and 5: Sample of a patient with HCC with heterozygous alleles of RASSF1A. Lanes 6 and 7: Sample of a patient with HCC with homozygous alleles of RASSF1A. RAASSF1A: Ras association domain family 1A; HCC: hepatocellular carcinoma

Aberrant methylation of RASSF1A was detected in 13 (65%) patients out of 20 patients with HCC (Figure 3). A significant association was detected between RASSF1A methylation and HCC with a p-value of 0.001. RASSF1A hypermethylation was completely absent in the control group (100%). In the cirrhotic group, RASSF1A aberrant hypermethylation was detected in only six (30%) patients out of 20 cirrhotic patients.

There was a statistically significant difference between the three groups regarding RASSF1A methylation status (p < 0.001). A significant statistical difference was detected between the group of cirrhotic patients and the group of healthy controls (p3 = 0.020) and the group of patients with HCC and controls (p2 < 0.001). We combined both homomethylated and hemimethylated status into one group of methylated RASSF1A. Hemimethylation implies that at a CpG site, only one strand of the DNA is methylated, while the other strand is unmethylated. A significant statistical difference was also detected between the group of cirrhotic patients and the group of healthy controls regarding the methylation status of RASSF1A (p3 = 0.020) and the group of patients with HCC and healthy controls (p2 < 0.001). We also detected a significant statistical difference between the group of patients with HCC and the group of cirrhotic patients (p1 = 0.027) (Table 2).

**Table 2:** Comparison between the studied groups according to RASSF1A methylation status. *: Statistically significant at p ≤ 0.05. χ^2^: chi-square test; MC: Monte Carlo test; F: F-test (one-way analysis of variance). **: Pairwise significance between groups was adjusted using the Monte Carlo test or the chi-square test. p1: P-value for comparing between the HCC group and the cirrhotic group. p2: P-value for comparing between the HCC group and the control group. p3: P-value for comparing between the cirrhotic group and the control group. RAASSF1A: Ras association domain family 1A; HCC: hepatocellular carcinoma

	HCC (n = 20)		Cirrhotic (n = 20)		Control (n = 20)		χ^2^	^MC^p
RASSF1A	No.	%	No.	%	No.	%		
Nonmethylated	7	35.0	14	70.0	20	100.0	20.462^*^	<0.001^*^
Hemimethylated	6	30.0	4	20.0	0	0.0		
Homomethylated	7	35.0	2	10.0	0	0.0		
Significance between groups	p1 =0.064, p2 < 0.001^**^, p3 = 0.020^**^							
Non-methylated	7	35.0	14	70.0	20	100.0	19.564^*^	<0.001^*^
Methylated	13	65.0	6	30.0	0	0.0		
Significance between groups	p1 = 0.027**, p2 < 0.001**, p3 = 0.020**							

We compared the distribution of demographic and clinical characteristics of included participants between two groups of patients with non-methylated and methylated RASSF1A. An insignificant difference existed in the proportion of males between both groups (85.7% vs. 92.3%, respectively, p = 1.000). We detected an insignificant statistical difference between non-methylated and methylated RASSF1A in the mean age of patients with HCC (57.14 ± 5.70 vs. 61.0 ± 5.58, respectively, p = 0.174). RASSF1A was non-methylated in four (57.1%) patients with a single HCC mass and in three (42.9%) patients with multiple HCC masses. It was methylated in seven (53.8%) patients with a single HCC mass and in six (46.2%) patients with multiple HCC masses (p = 1.000). The median size of HCC mass as well as different categories of BCLC staging system whether very early stage, early stage (A), intermediate stage (B), advanced stage (C), or terminal stage (D) did not differ significantly between RASSF1A methylation status (p = 0.449, p = 0.958, respectively). Statistically, an insignificant difference existed in the mean value of HCV viral load between non-methylated and methylated RASSF1A in HCC and cirrhotic groups (p = 0.589, p = 0.249, respectively).

Serum levels of AFP ranged from 3.50 to 1,044.0 ng/mL with a median value of 8.10 ng/mL in seven HCC patients with non-methylated RASSF1A alleles. It ranged from 3.50 to 1,075.0 ng/mL with a median value of 12.5 ng/mL in 13 HCC patients with methylated RASSF1A alleles. There was no statistically significant difference between the methylation status of RASSF1A and serum AFP level in patients with HCC (p = 0.874) (Table 3).

**Table 3:** Correlation between RAASSF1A methylation and other parameters in the HCC group. χ^2^: chi-square test; FE: Fisher exact test; t: Student’s t-test; Z: Z for Mann-Whitney test. RAASSF1A: Ras association domain family 1A; HCC: hepatocellular carcinoma; BCLC: Barcelona Clinic Liver Cancer; AFP: alpha-fetoprotein; HCV: hepatitis C virus; PCR: polymerase chain reaction

	RAASSF1A non-methylated (n = 7)		RAASSF1A methylated (n = 13)		Test of significance	P
Sex	No.	%	No.	%	χ^2^	^FE^p
Male	6	85.7	12	92.3	0.222	1.000
Female	1	14.3	1	7.7		
Age (years), mean ± SD	57.14 ± 5.70		61.0 ± 5.58		t = 1.417	0.174
Tumor size					Z	P
Median (minimum–maximum)	2.0 (1.0–4.50)		1.70 (0.50–3.20)		0.757	0.449
Number of hepatic masses	No.	%	No.	%	χ^2^	^FE^p
Single	4	57.1	7	53.8	0.020	1.000
Multiple	3	42.9	6	46.2		
BCLC stage	No.	%	No.	%	χ^2^	^MC^p
Very early stage (0)	1	14.3	3	23.1	1.881	0.958
Early stage (A)	3	42.9	4	30.8		
Intermediate stage (B)	2	28.6	3	23.1		
Advanced stage (C)	1	14.3	1	7.7		
Terminal stage (D)	0	0.0	2	15.4		
AFP (ng/mL)					Z	P
Minimum–maximum	3.50–1044.0		(3.50–1075.0)		0.159	0.874
Median	30.10		12.50			
HCV-PCR in HCC group	(n = 7)		(n = 13)		T	P
Mean ± SD	2.16 ± 0.75		2.32 ± 0.53		0.549	0.589
HCV-PCR in Cirrhotics	(n = 14)		(n = 6)		1.192	0.249
Mean ± SD	2.33 ± 0.55		2.68 ± 0.76			

The assessment of the methylation status of RASSF1A had a sensitivity of 65.0%, specificity of 70.0%, PPV of 68.42%, NPV of 66.67%, and accuracy of 67.50% in detecting HCC in cirrhotic patients (Figure 1). The assessment of the methylation status of RASSF1A showed 65.0% sensitivity, 100% specificity, 100% PPV, 74.07% NPV, and 82.50% accuracy in the detection of HCC in the control group (Figure 2).

ROC curve analysis was performed to evaluate the sensitivity and specificity of the combination of serum AFP and assessment of RASSF1A methylation status in the detection of HCC in cirrhotic patients. At a cut-off value of 17 ng/mL, this combination had a sensitivity of 65.0%, specificity of 70.0%, PPV of 68.42%, NPV of 66.67%, and accuracy of 67.50% (AUC = 0.685) (Figure 1). In addition, ROC curve analysis was performed to evaluate the sensitivity and specificity of the combination of serum AFP and the assessment of RASSF1A methylation status in the detection of HCC in normal persons (control group). At a cut-off value of 6 ng/mL, this combination had a sensitivity of 85.0%, specificity of 100.0%, PPV of 100.0%, NPV of 86.96%, and accuracy of 92.50%. The AUC was 0.945 (Figure 2).

## Discussion

HCC is considered the most common type of primary liver cancer [19]. Serum AFP evaluation is no longer recommended as a serological screening tool. Its assessment does not add much value to the efficacy of the screening program rather than being an additional economic burden on patients that can influence their compliance with the screening program. The usage of CT and/or MRI in HCC screening is not supported except in certain circumstances [4,20].

Detection of aberrant DNA methylation (hypo/hypermethylation) is a very promising non-invasive marker for the early detection of HCC as well as a prognostic marker for HCC progression for the following reasons: it is detected in chronic liver disease and/or cirrhotic liver even before HCC development; it is a positive signal not detected in normal cells, thus there would not be any doubts in results when there is a mixture of normal and malignant cells in the sample; the stability of aberrant DNA methylation allows its identification from plasma or other body fluids of the patients where the circulating tumor cells (CTCs) are present [6,7].

RASSF1A has been implicated in the regulation of a wide range of physiological functions that are essential for normal growth control and apoptosis. Many studies have proven RASSF1A aberrant promoter hypermethylation in different cancer types that causes its transcriptional silencing as a tumor suppressor gene which enhances carcinogenesis [9].

In this study, we found that the aberrant methylation of RASSF1A was detected in 13 (65%) patients out of 20 patients with HCC. A statistically significant difference was detected between the group of patients with HCC and the group of cirrhotic patients and the control group regarding RASSF1A methylation status with a p-value <0.001.

In agreement with our results, Dong et al. reported that serum RASSF1A methylation was detected more frequently in patients with HCC. It was detected in 64.2% of patients with HCC. Serum RASSF1A methylation in patients with HCC was significantly higher than in patients with cirrhosis or chronic hepatitis B (p < 0.001) [17]. Several studies have reported similar results using both MSP and MethyLight as methods of detection [9,17,21,22].

Our results were different from those reported by Mohamed et al. [10]. In their study conducted on 40 patients with HCC, real-time PCR after digestion with a methylation-sensitive restriction enzyme was employed. They detected aberrant RASSF1A hypermethylation in the sera of 36 (90%) patients with HCC. The mean level of methylated RASSF1A in patients with HCC was significantly higher than the level of the control group (p = 0.001) and cirrhotic patients (p = 0.001). This discrepancy in the results can be attributed to the different methods of detection used. MSP is a qualitative method of detection that lacks the advantage of a more sensitive quantitative method of detection as real-time PCR after digestion with a methylation-sensitive restriction enzyme. Contrary to our study, Hu et al. [23] identified aberrant serum RASSF1A hypermethylation by MSP. The aberrant hypermethylation of RASSF1A was detected in 14 (40%) out of 35 patients with HCC.

Zekri et al. [24] found aberrant RASSF1A hypermethylation in 100% of patients in their study conducted on 31 patients with HCC using MSP. The difference between the results of both studies and the current one may be due to their smaller sample size.

Our study is supported by an interesting Brazilian study that was conducted on 41 liver tissue samples of non-cirrhotic, cirrhotic, and HCC tissue samples and measured the DNA methylation levels of RASSF1A promoter regions by the highly sensitive pyrosequencing quantitative technology. The mean methylation rates in RASSF1A were 16.2% in non-cirrhotic, 26.1% in cirrhotic, and 59.1% in HCC tissues. No statistical difference in methylation levels was detected between cirrhotic and non-cirrhotic tissues (RASSF1A, p = 0.165). However, they observed a highly significant difference in methylation levels between HCC and non-HCC tissues. They found no association between tumor size and methylation levels, as seen in this study. They recommended the use of non-invasive approaches that measure hypermethylation of the circulating cell-free DNA in serum/plasma [25].

When discussing the relationship between the methylation status of RAASSF1A and other parameters (demographic, clinicopathological, and laboratory) in the HCC group in our study, we found similar results in the pooled analyses conducted by Xu et al. [26]. This meta-analysis included 44 studies that reported the relationship of the promoter methylation of RASSF1A with HCC or the associated clinicopathological characteristics in patients with HCC, studies that investigated the promoter methylation levels of RASSF1A in both tissues and blood, case-control studies that regarded HCC and non-HCC people as cases (as confirmed from liver tissues and peripheral blood), and studies that reported the exact frequency of RASSF1A methylation in both cases and controls. They found RASSF1A promoter hypermethylation was remarkably related to tumor size (p = 0.028) but not significantly associated with sex (p = 0.094), age (p = 0.152), HCV infection (p = 0.928), level of AFP (p = 0.657), and tumor number (p = 0.410).

Contrary to our results, Araujo et al. [25] observed higher levels of RASSF1A methylation in HCC tissues of the younger group (<40 years) which could be explained by observing that all HCC patients under 40 years of age in their study were chronically infected with HBV, possibly infected a long time ago through vertical transmission. On the other hand, all our patients had HCV-related HCC, and an insignificant statistical difference was found between methylated RASSF1A and the mean age.

Our study showed that RASSF1A aberrant hypermethylation could detect HCC in cirrhotic patients with 65% sensitivity, 70% specificity, 68.42% PPV, 66.67% NPV, and 67.5% accuracy. The diagnostic performance of RASSF1A in detecting HCC in normal controls revealed 65% sensitivity, 100% specificity, 100% PPV, 74.07% NPV, and 82.5% accuracy. The study by Huang et al. [27] agreed with our results. Methylation-sensitive restriction enzyme-based quantitative PCR was used to evaluate RASSF1A methylation status in the plasma of 72 patients with HCC, 32 benign hepatic disease, and 41 normal controls. RASSF1A methylation could detect HCC with a sensitivity of 63.9%, a specificity of 94.6%, a PPV of 95.5%, and an NPV of 57.4%.

We found no significant statistical difference between serum RASSF1A methylation status and serum level of AFP in patients with HCC. Our results were supported by Xu et al. [28] and Yeo et al. [14]. They did not find any significant statistical difference between RASSF1A methylation status and serum AFP level in patients with HCC. The diagnostic performance of the combination of the assessment of RASSF1A methylation status and serum AFP level in the detection of HCC in cirrhotic patients yielded 65% sensitivity, 70% specificity, 68.42% PPV, 66.67% NPV, and 67.5% accuracy. Dong et al. [17] found that the combination of serum AFP level and serum RASSF1A methylation status had a sensitivity of 80.9%, 93.4% specificity, and AUC of 0.876.

Study strengths

To avoid selection bias, all patients were recruited from the inpatient ward and the outpatient clinic of the Hepatobiliary Unit, Department of Internal Medicine at the university hospital. The control group participants were recruited from the minor surgery clinic at the same hospital. All participants in this study were subjected to full history taking, physical examination, and radiological assessment of the abdomen. Laboratory investigations for liver and kidney functions, PT, AFP, viral markers, and CBC were performed. Finally, the methylation status of RASSF1A was assessed using MSP, which is a useful tool for qualitative DNA methylation analysis with multiple advantages, including ease of design, sensitivity in the ability to detect small quantities of methylated DNA, and the ability to rapidly screen a large number of samples without the need for expensive laboratory reagents and equipment.

Study limitations

Given the case-control study design, HCC prevalence needs to be considered while calculating PPV and NPV. However, despite this difference in the study design, Dong et al. [15] and Azab et al. [19] also reported similar results to our study. One of the limitations of this study was the small sample size and small subgrouping between the studied groups. Therefore, further studies should be replicated at multiple centers with a large sample size.

## Conclusions

There was a significant increase in the methylation of RASSF1A in the group of patients with HCC on top of HCV-related liver cirrhosis in comparison to the group of patients with HCV-related liver cirrhosis and the control group. MSP is a useful, sensitive, rapid, and qualitative method for the assessment of the methylation status of different tumor suppressor genes in the peripheral blood of patients with HCC. Assessment of the methylation status of the promoter of RASSF1A in the peripheral blood of patients with HCC may be considered a new non-invasive marker that can aid in the early diagnosis of HCC. The assessment of the methylation status of RASSF1A has a better diagnostic performance than AFP in the diagnosis of HCC. Larger studies are recommended to confirm the ability of the methylation status of RASSF1A assessment in the diagnosis of HCC. The combination of MSP with a quantitative method such as MethyLight or sequencing is recommended in the assessment of the methylation status of RASSF1A for further confirmation of its diagnostic power in HCC detection.
